# Supplementary material for: Does route matter? Impact of route of oxytocin administration on postpartum bleeding: A double-blind, randomized controlled trial
Source: PLoS One. 2019 Oct 1;14(10):e0222981. doi: 10.1371/journal.pone.0222981 (PMC6772050; doi:10.1371/journal.pone.0222981)
Supplement: S1 Checklist — (DOC) [file pone.0222981.s001.doc]

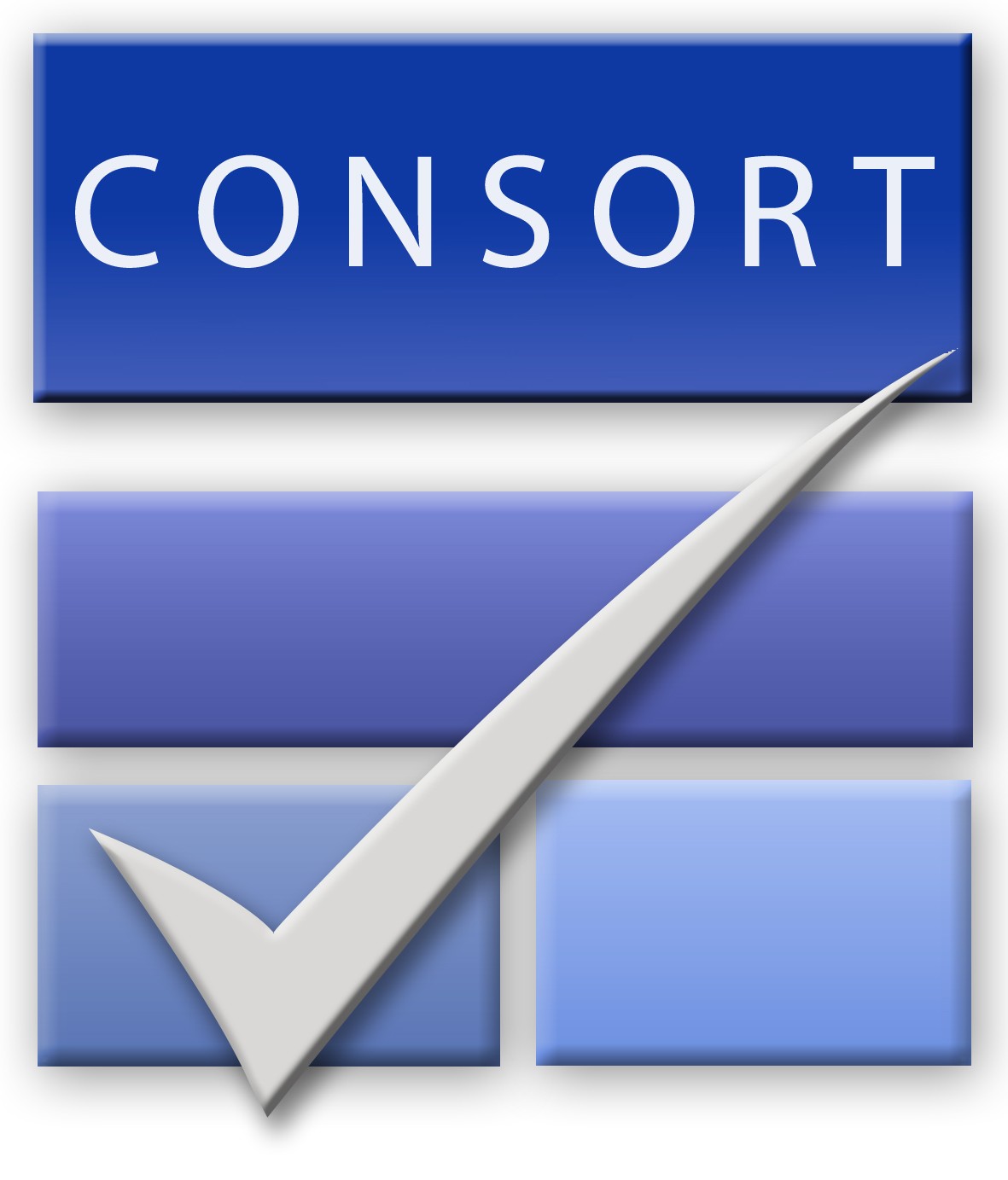
CONSORT 2010 checklist of information to include when reporting a randomised trial*

| Section/Topic | Item No | Checklist item | Reported on page No |
| --- | --- | --- | --- |
| Title and abstract | | | |
|  | 1a | Identification as a randomised trial in the title | See title,  pg. 1 |
|  | 1b | Structured summary of trial design, methods, results, and conclusions (for specific guidance see CONSORT for abstracts) | See abstract, pg. 2 |
| Introduction | | | |
| Background and objectives | 2a | Scientific background and explanation of rationale | See Intro,  pg. 3-4 |
|  | 2b | Specific objectives or hypotheses | See Intro,  pg. 4 |
| Methods | | | |
| Trial design | 3a | Description of trial design (such as parallel, factorial) including allocation ratio | See Methods, pg. 4 |
|  | 3b | Important changes to methods after trial commencement (such as eligibility criteria), with reasons | n/a |
| Participants | 4a | Eligibility criteria for participants | See Methods, pg. 4 |
|  | 4b | Settings and locations where the data were collected | See Methods, pg. 4 |
| Interventions | 5 | The interventions for each group with sufficient details to allow replication, including how and when they were actually administered | See Methods, pg. 5 |
| Outcomes | 6a | Completely defined pre-specified primary and secondary outcome measures, including how and when they were assessed | See Methods, pg. 6 |
|  | 6b | Any changes to trial outcomes after the trial commenced, with reasons | n/a |
| Sample size | 7a | How sample size was determined | See Methods, Pg. 7-8 |
|  | 7b | When applicable, explanation of any interim analyses and stopping guidelines | n/a |
| Randomisation: |  |  |  |
| Sequence generation | 8a | Method used to generate the random allocation sequence | See Methods, pg. 5 |
|  | 8b | Type of randomisation; details of any restriction (such as blocking and block size) | See Methods, pg. 5 |
| Allocation concealment mechanism | 9 | Mechanism used to implement the random allocation sequence (such as sequentially numbered containers), describing any steps taken to conceal the sequence until interventions were assigned | See Methods, pg. 5 |
| Implementation | 10 | Who generated the random allocation sequence, who enrolled participants, and who assigned participants to interventions | See Methods, pg. 5 |
| Blinding | 11a | If done, who was blinded after assignment to interventions (for example, participants, care providers, those assessing outcomes) and how | See Methods, pg. 5 |
|  | 11b | If relevant, description of the similarity of interventions | n/a |
| Statistical methods | 12a | Statistical methods used to compare groups for primary and secondary outcomes | See Methods, pg. 8 |
|  | 12b | Methods for additional analyses, such as subgroup analyses and adjusted analyses | n/a |
| Results | | | |
| Participant flow (a diagram is strongly recommended) | 13a | For each group, the numbers of participants who were randomly assigned, received intended treatment, and were analysed for the primary outcome | See results and figure 1 |
|  | 13b | For each group, losses and exclusions after randomisation, together with reasons | See results and figure 1 |
| Recruitment | 14a | Dates defining the periods of recruitment and follow-up | See Results, pg. 8, para 1 |
|  | 14b | Why the trial ended or was stopped | n/a |
| Baseline data | 15 | A table showing baseline demographic and clinical characteristics for each group | See results and tables 1-2 |
| Numbers analysed | 16 | For each group, number of participants (denominator) included in each analysis and whether the analysis was by original assigned groups | See results and table 3 |
| Outcomes and estimation | 17a | For each primary and secondary outcome, results for each group, and the estimated effect size and its precision (such as 95% confidence interval) | See results and table 3 |
|  | 17b | For binary outcomes, presentation of both absolute and relative effect sizes is recommended | See results and table 3 |
| Ancillary analyses | 18 | Results of any other analyses performed, including subgroup analyses and adjusted analyses, distinguishing pre-specified from exploratory | See results on pg. 14 |
| Harms | 19 | All important harms or unintended effects in each group (for specific guidance see CONSORT for harms) | See results, last para |
| Discussion | | | |
| Limitations | 20 | Trial limitations, addressing sources of potential bias, imprecision, and, if relevant, multiplicity of analyses | See discussion, pg. 18-19 |
| Generalisability | 21 | Generalisability (external validity, applicability) of the trial findings | See discussion, pg. 18-19 |
| Interpretation | 22 | Interpretation consistent with results, balancing benefits and harms, and considering other relevant evidence | See discussion pg. 16-17 |
| Other information | | |  |
| Registration | 23 | Registration number and name of trial registry | See Methods, pg. 4 |
| Protocol | 24 | Where the full trial protocol can be accessed, if available | Supplementary file |
| Funding | 25 | Sources of funding and other support (such as supply of drugs), role of funders | Specified online at submission |

*We strongly recommend reading this statement in conjunction with the CONSORT 2010 Explanation and Elaboration for important clarifications on all the items. If relevant, we also recommend reading CONSORT extensions for cluster randomised trials, non-inferiority and equivalence trials, non-pharmacological treatments, herbal interventions, and pragmatic trials. Additional extensions are forthcoming: for those and for up to date references relevant to this checklist, see [www.consort-statement.org](http://www.consort-statement.org/).
