# Supplementary figures and images for: Does route matter? Impact of route of oxytocin administration on postpartum bleeding: A double-blind, randomized controlled trial
Source: PLoS One. 2019 Oct 1;14(10):e0222981. doi: 10.1371/journal.pone.0222981 (PMC6772050; doi:10.1371/journal.pone.0222981)

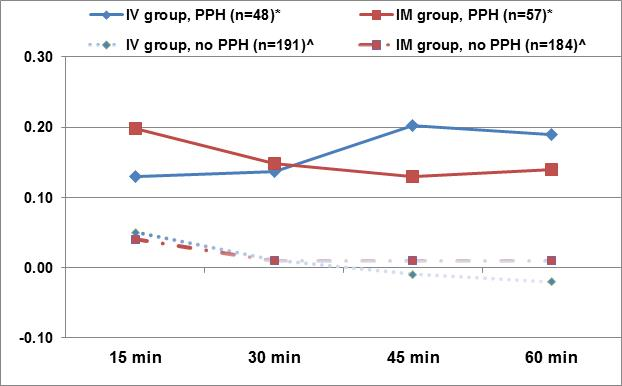

Supplement: S1 Fig — *Among the PPH cases, the median change in SI from pre-delivery to 15-minutes postpartum was higher in the IM group (median Δ 0.20, IQR 0.08, 0.33), compared to the IV infusion group (median Δ 0.13 IQR -0.02, 0.22; p = 0.048); change in SI between study groups were comparable at all other time intervals (p>0.05). ^Among the non-PPH cases, the median changes in SIs were not statistically different between study groups at any time postpartum (p>0.05). (TIF) [file pone.0222981.s002.tif]

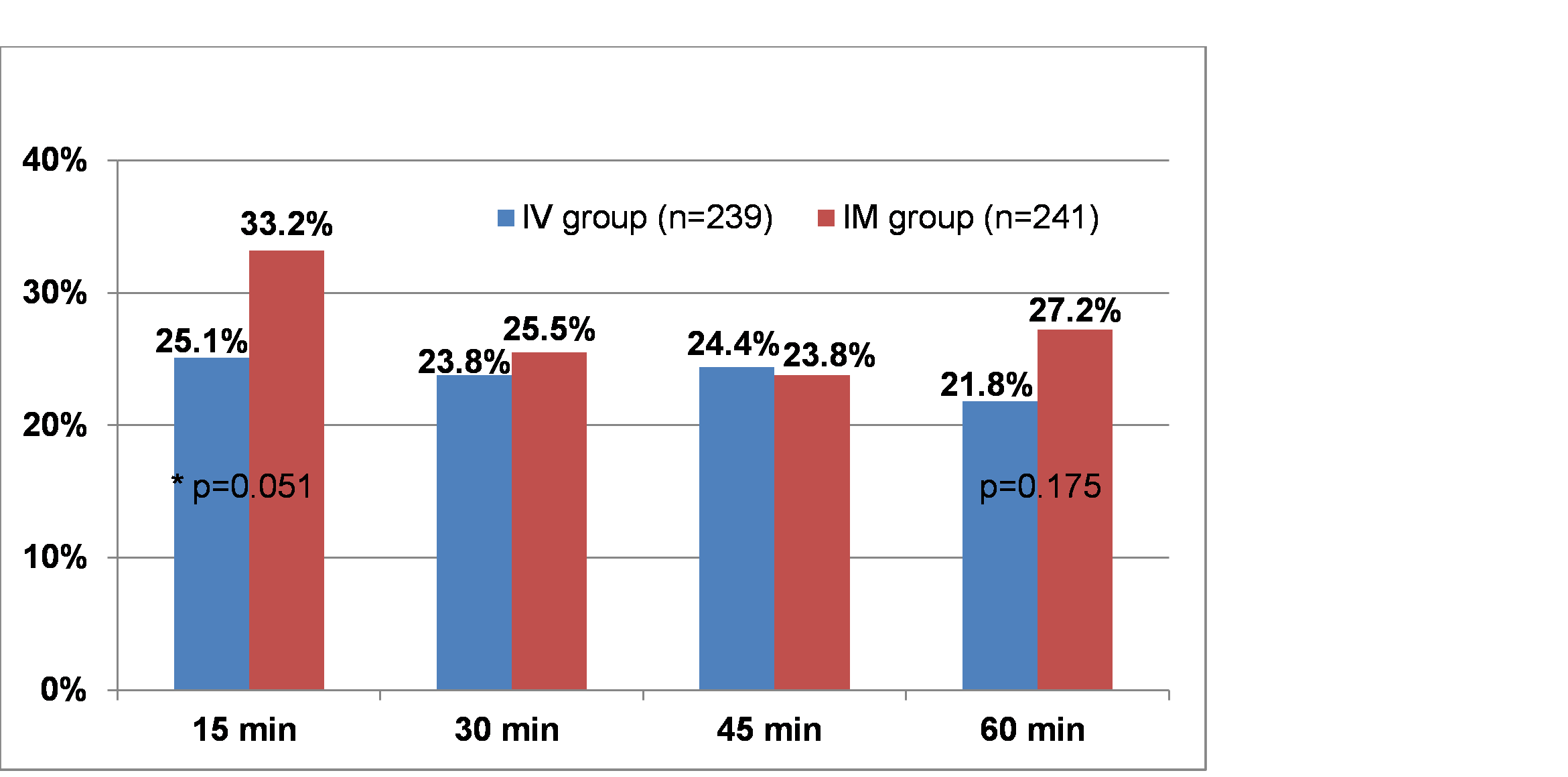

Supplement: S2 Fig — (TIF) [file pone.0222981.s003.tif]
