# Supplementary material for: Does route matter? Impact of route of oxytocin administration on postpartum bleeding: A double-blind, randomized controlled trial
Source: PLoS One. 2019 Oct 1;14(10):e0222981. doi: 10.1371/journal.pone.0222981 (PMC6772050; doi:10.1371/journal.pone.0222981)
Supplement: S1 Protocol — (DOC) [file pone.0222981.s004.doc]

**Número de protocolo**: 3008

**Título del protocolo:** La administración de oxitocina intravenosa versus intramuscular y su relación con el sangrado posparto y otros signos clínicos: un estudio aleatorizado y controlado por placebo

**Periodo propuesto para la realización del proyecto:** el primero de septiembre, 2016 al 30 de abril, 2017

**Investigadora principal**

Dra. Beverly Winikoff, Gynuity Health Projects, Estados Unidos

**Co-investigadores:**

# Jill Durocher, Gynuity Health Projects, Estados Unidos

# Ilana Dzuba, Gynuity Health Projects, Estados Unidos

# Dr. Guillermo Carroli, Centro Rosarino de Estudios Perinatales (CREP)

# Co-investigadores del lugar del estudio: *Hospital J.R. Vidal, Corrientes*

# Preparado por:

# Gynuity Health Projects

# 15 East 26^th^ Street, Suite 801

# NY, NY 10010

# Estado Unidos

**Antecedentes**

La hemorragia posparto es una importante causa de mortalidad materna en todo el mundo y muy comúnmente es el resultado de una atonía uterina, es decir, de los casos en que el útero no se contrae para evitar la pérdida de sangre después del parto. Se recomienda el manejo activo de la tercera etapa del parto (MATEP) tanto en escenarios bien equipados, como de bajos recursos por su eficacia para prevenir la hemorragia posparto derivada de la atonía uterina. La administración de un uterotónico sintético estimula el músculo liso del útero, para mejorar el tono muscular y es el componente más importante del MATEP, que se practica en muchas partes del mundo. La oxitocina se reconoce universalmente como el uterotónico de elección para esa indicación. Estudios comparativos sobre el uso de oxitocina sola o en combinación con otros componentes del MATEP muestran una disminución en el riesgo de sangrado y de hemorragia posparto (HPP) .

En la literatura disponible sobre el uso de la oxitocina para la prevención de la HPP se encuentran numerosos estudios que discrepan en lo relacionado a su forma de administración (e.g. vía, dosis y tiempos) . Por ejemplo, en algunos estudios la oxitocina se administra por vía intravenosa (IV) , mientras que en otros se administra por vía intramuscular (IM) . El supuesto subyacente ha sido que las distintas vías de administración no poseen efectos diferenciales en la magnitud de la pérdida de sangre en el posparto . Sin embargo, los resultados de un estudio realizado en 1972 sobre los niveles de oxitocina en la circulación comparando inyecciones IV, IM y subcutáneas de Sintometrina [5 UI de oxitocina y 0.5 mg de ergometrina) en una pequeña muestra de mujeres (n=26) indicaron que los niveles plasmáticos de oxitocina se elevan más rápidamente cuando se administra por vía IV y alcanzan un pico mucho más alto que con la administración IM .

La variabilidad en la vía de administración de la oxitocina observada en la literatura se refleja también en la práctica clínica y en las guías de manejo, y no parece existir acuerdo o procedimiento estándar universal para la provisión de oxitocina profiláctica en la tercera etapa del parto. En algunas guías de manejo se especifica la administración IM, que se considera la vía más razonable en servicios de atención primaria y otros escenarios donde la inyección IV no resulta viable debido a las habilidades y permisos requeridos y porque complica la movilidad y la atención de las pacientes . En otras guías de manejo, no se establece una vía de administración específica o se recomiendan las dos vías como alternativas similares .

Muy probablemente, gran parte de las variaciones observadas en los protocolos clínicos respecto a las formas de administración de la oxitocina se pueda atribuir a datos inadecuados o en disputa sobre la farmacocinética y la farmacodinámica de la oxitocina. El tiempo requerido para alcanzar la concentración plasmática estable depende de la vida media del medicamento; cuanto más prolongada, más tiempo se requerirá para alcanzar el nivel estable. Usualmente se reporta que la vida media de la oxitocina es de aproximadamente tres minutos, aunque algunos autores reportan una vida media de hasta 20 minutos. Independientemente de la vía de administración, la oxitocina actúa rápidamente. La sensibilidad uterina al medicamento aumenta a lo largo del embarazo, debido a la mayor concentración de receptores de oxitocina con el aumento de la edad gestacional. Después de la inyección IV, el inicio de la acción es casi inmediato, dentro del primer minuto, y se inicia un poco más lentamente, a los 2-4 minutos, con la inyección IM. Con la infusión IV, la respuesta uterina es gradual y alcanza el estado de concentración estable después de 15-40 minutos; contracciones ininterrumpidas, aunque la velocidad de inyección caracterizada por el diámetro de la aguja, el tamaño del catéter y la cantidad del líquido de dilución pueden influir en el tiempo requerido para alcanzar dicha concentración estable.

Si bien no se han comparado rigurosamente la administración IV e IM de oxitocina en la tercera etapa del parto, los autores de distintos artículos cuestionan la posibilidad de que la vía de administración produzca algún efecto, en particular en lo relacionado a la velocidad de inyección y cómo influye en la prevención de la pérdida de sangre posparto. Un análisis secundario de un ensayo clínico a gran escala en el cual las participantes recibieron oxitocina profiláctica en la tercera etapa del parto sugiere que la vía de administración sí importa, en especial cuando la oxitocina es el único componente del MATEP que se proporciona . La pérdida promedio de sangre en el posparto fue de 336 ml en mujeres que recibieron sólo oxitocina profiláctica por vía IM en la tercera etapa del parto (n=2845) y 60 ml menor (277 ml) en mujeres que recibieron sólo oxitocina profiláctica por vía IV (n=785). Las participantes que recibieron oxitocina profiláctica IV presentaron menor riesgo de una pérdida de sangre ≥700 ml en el posparto que las mujeres que recibieron oxitocina IM (RM 0.24, IC 95% 0.12-0.51). La prevalencia de perdida de ese volumen de sangre fue de 0.5% en vez de 2.1%. De las 785 mujeres que recibieron oxitocina IV, la gran mayoría recibió una inyección intravenosa en bolo. Otro estudio reveló que la administración de un bolo IV de oxitocina produjo una menor pérdida de sangre promedio estimada y de significancia estadística, comparada con la infusión IV de oxitocina (358 ml vs 424 ml, p=0.029) . En ese estudio, no se midió la sangre en un recipiente calibrado y la pérdida de sangre la calcularon los proveedores mediante una inspección visual; sin embargo, da lugar a cuestionar si el bolo IV y la infusión IV son tan similares como se supone.

En muchos lugares se considera que la oxitocina es un medicamento seguro cuando se le utiliza de forma profiláctica para la prevención de la HPP. Rara vez se observan efectos secundarios en mujeres con partos vaginales, aunque se ha reportado la presencia de náuseas y vómitos. En un estudio donde se comparaba la hemodinamia materna tras la administración de un bolo o infusión de oxitocina en la tercera etapa del parto después de un nacimiento vaginal, se encontró que la administración de un bolo de oxitocina de 10 UI no se asocia a respuestas hemodinámicas adversas y se puede administrar sin riesgos en la tercera etapa del parto a mujeres con acceso intravenoso como profilaxis para prevenir la hemorragia posparto.

En el presente protocolo, describimos un estudio controlado y aleatorizado que busca evaluar el efecto de la vía de administración de 10 UI de oxitocina en la pérdida promedio de sangre en hospitales de nivel terciario, donde la política de atención estándar es la administración de oxitocina profiláctica en la tercera etapa del parto. (La dosis de oxitocina profiláctica varía de 5 a 40 UI, pero dado que el uso de 10 UI es más común en varios modos de administración y es el recomendado por la Organización Mundial de la Salud, estandarizaremos una dosis de 10 UI para todos los grupos de tratamiento.) En nuestro análisis, se documentará y controlará la práctica de otros componentes del MATEP. También evaluaremos los efectos secundarios y eventos adversos asociados a cada vía de administración, incluida la pérdida de sangre.

Los resultados de ese estudio llenarán un vacío en la literatura en cuanto a determinar si la vía de administración de la oxitocina durante la tercera etapa del parto afecta de alguna manera la pérdida de sangre. Además, los resultados de este estudio podrían tener implicancias importantes para el uso de los uterotónicos no parenterales profilácticos en la tercera etapa del parto, tales como el misoprostol, un análogo de la prostaglandina E1 que es eficaz en la reducción de la HPP moderada y severa. Las guías actuales recomiendan la administración de oxitocina (por infusión IV o inyección IM) en vez de misoprostol. Sin embargo, la diferencia en eficacia, favoreciendo la vía IV de administración de la oxitocina para detener la hemorragia puede sugerir un rol más importante para el misoprostol en lugares en donde la oxitocina está disponible, pero la vía IV no es factible.

Además de los estudios de investigación actualmente en curso sobre el manejo de la HPP, existe la necesidad de evaluar nuevos indicadores clínicos que pudieran facilitar el diagnóstico y tratamiento tempranos de la HPP. Por ahora, una intervención clínica oportuna y apropiada depende en gran medida de la habilidad del proveedor de salud de evaluar visualmente la pérdida de sangre para determinar la pertinencia del tratamiento. Se ha demostrado que calcular visualmente la pérdida de sangre en el posparto resulta difícil e impreciso en varios escenarios de atención del parto . Más aun, persiste la incertidumbre respecto a la cantidad de sangre perdida que es de mayor importancia para predecir resultados maternos severos en mujeres que presentan sangrado excesivo. Algunos signos y síntomas clínicos, tales como palidez, debilidad, palpitaciones, taquicardia, confusión y oliguria, entre otros, se han correlacionado con el volumen de pérdida de sangre y podrían servir para iniciar una intervención clínica para el manejo de la HPP . De acuerdo a una revisión sistemática de la literatura para evaluar la relación que existe entre signos y síntomas clínicos y la pérdida de sangre y los resultados maternos, un índice de shock, definido por la razón entre la frecuencia cardíaca y la presión arterial sistólica, puede servir para evaluar la severidad de la pérdida de sangre. Antes del año 2013, la mayoría de los artículos revisados reportaron que existe una asociación entre frecuencia cardiaca, presión arterial y/o índice de shock y la pérdida de sangre, pero sólo unos pocos se realizaron en poblaciones obstétricas, donde los cambios hemodinámicos producidos por el embarazo podrían generar una relación diferente entre signos clínicos y pérdida de sangre, razón por la cual se requiere mayor investigación para valorar la relación entre el índice de shock y la gravedad de la pérdida de sangre en el posparto inmediato. Estudios retrospectivos recientemente publicados que analizaron la relación entre la HPP severa y los signos vitales sugieren que medidas altas de índice de shock (e.g. un índice de shock ≥ 0.9 o ≥ 1.7) pueden ayudar a identificar a las mujeres que necesitan atención urgente .

**Objetivos de investigación**

Objetivos primarios

- Evaluar si la oxitocina profiláctica administrada a mujeres en la tercera etapa del parto por medio de infusión IV conlleva una menor pérdida de sangre promedio que la inyección IM del medicamento.
- Evaluar el efecto de la administración de oxitocina profiláctica en mujeres en la tercera etapa del parto mediante infusión IV comparada con la administración IM de oxitocina en la incidencia de hemorragia posparto(pérdida de sangre ≥ 500 ml).

Objetivos secundarios

- Comparar el efecto de la infusión IV y la administración IM de oxitocina en la proporción de mujeres que experimentan pérdida de sangre ≥ 1000 ml, como así también en efectos secundarios y cambios en los niveles de hemoglobina antes y después del parto.
- Determinar la relación que existe entre los cambios posparto en el índice de shock y la severidad de la pérdida de sangre y otros resultados de la HPP.

El estudio proporcionará respuestas a las siguientes preguntas de investigación:

1. ¿La administración de oxitocina vía infusión IV es más efectiva que la administración IM del medicamento para reducir la pérdida de sangre posparto cuando se proporciona como parte del MATEP?
2. ¿La diferencia promedio entre los niveles de hemoglobina antes y después del parto es menor con la infusión IV que con la administración IM?
3. ¿Los perfiles de los efectos secundarios varían entre la infusión IV y la administración IM de oxitocina?
4. ¿El índice de shock podría representar un indicador útil para evaluar la severidad de la pérdida de sangre en la primera hora del puerperio y para iniciar una intervención clínica para el manejo de la HPP?

**Medidas de resultados**

Medidas de resultados primarios:

- Pérdida promedio de sangre (ml)
- Proporción de mujeres que experimenta una pérdida de sangre posparto ≥ 500 ml

Medidas de resultados secundarios:

- Proporción de mujeres con pérdida de sangre posparto ≥ 1000 ml
- Cambio de los niveles de hemoglobina entre la etapa previa al parto y en el posparto
- Tiempo requerido para la expulsión de la placenta
- Administración de oxitocina adicional, otros uterotónicos u otras intervenciones como transfusión sanguínea e histerectomía
- Efectos secundarios hasta una hora después del parto
- Medidas de presión arterial y frecuencia cardiaca durante la primera hora del posparto.

**Descripción de los centros de estudio**

*Corrientes, Argentina:* El Hospital J.R. Vidal es una institución pública de atención terciaria ubicada en la ciudad de Corrientes, capital de la Provincia del mismo nombre. La provincia se encuentra en el noreste de Argentina y limita con los países de Paraguay, Brasil y Uruguay. En el año 2012, el Hospital Vidal atendió un total de 3.080 nacimientos. Aproximadamente, un 30% de los nacimientos fueron por cesáreas. La oxitocina se consigue fácilmente en dichos hospitales y se administra 10 UI de oxitocina de forma rutinaria durante la tercera etapa del parto en todas las mujeres que tienen un nacimiento. En los casos de HPP, se administran oxitócicos adicionales por una vía intravenosa según las normas hospitalarias.  El equipo local ha demostrado un gran interés en participar en la investigación de HPP. Su personal ha participado en otro ensayo clínico con la colaboración de Gynuity Health Projects para estudiar el tema de la HPP; asimismo, el personal cuenta con amplia experiencia en la medición objetiva de la pérdida de sangre en el posparto, en el diagnóstico y tratamiento de la HPP y en la medición de hemoglobina. Además, los resultados del estudio anterior revelaron una tasa alta de HPP (≥ 500 ml) en este hospital, alrededor de 18%. Este hospital también tiene una importante experiencia previa en investigaciones de intervenciones en colaboración con el Dr. Guillermo Carroli, Director del Centro Rosarino de Estudios Perinatales (CREP), el cual es un centro colaborador de la Organización Mundial de la Salud.

**Explicación sobre el tamaño de la muestra**

*Argentina:* Las medidas de resultados primarios para este estudio son: (1) la pérdida promedio de sangre y (2) la proporción de mujeres que experimenta pérdida de sangre ≥ 500 ml.

El estudio se diseñó para detectar una diferencia de 50 ml en la pérdida promedio de sangre después de administrar oxitocina en forma profiláctica por medio de infusión IV e inyección IM. En la literatura disponible, se ha demostrado que una diferencia de 50 ml se traduce en una disminución aproximada del 50% en las tasas de HPP (≥500 ml) . De acuerdo con el supuesto de que la oxitocina profiláctica administrada por vía intravenosa produce una pérdida de sangre promedio de 275 ml (DE 175) [29], calculamos que se requeriría un total de 257 mujeres para este ensayo de superioridad para detectar una diferencia de 50 ml en la pérdida de sangre promedio entre los dos grupos de estudio, con una potencia del 80% y un nivel de significancia de 0.05.

Un estudio anterior llevado a cabo en el Hospital Vidal acerca de la HPP  había documentado una tasa de la pérdida de sangre ≥500 ml de 18% entre mujeres que recibieron oxitocina profiláctica (10 UI) durante la tercera etapa del parto y a quienes habían tenido su sangrado posparto medido a través del uso de un recipiente calibrado. De acuerdo con el supuesto de que la oxitocina profiláctica administrada por vía intravenosa produce un reducción importante clínicamente de al menos 50% en la proporción de mujeres con una pérdida de sangre de ≥500 ml ~~,~~ calculamos que se requeriría un total de 442 mujeres (partos vaginales) (221 por grupo) para este ensayo de superioridad para comparar una tasa de 18% de HPP con la administración IM de oxitocina y una tasa de 9% de HPP por medio de infusión IV, con una potencia del 80% y un nivel de significancia de 0.05. El tamaño de muestra se incrementará un 10% para dar cuenta de pérdidas en el seguimiento o la falta de mediciones de sangrado posparto para la evaluación de los dos resultados primarios. Por consiguiente, se reclutará en el estudio de Argentina **una muestra total de 486 mujeres (243 por grupo)**. Este tamaño de la muestra resulta adecuado para detectar una diferencia de 50 ml en la pérdida promedio de sangre después de la administración profiláctica de oxitocina por medio de infusión IV e inyección IM en la tercera etapa del parto.

**Descripción del estudio de investigación**

Este estudio doble ciego, controlado y aleatorizado se llevará a cabo en hospitales cuyas políticas estipulen que todas las parturientas deben recibir 10 UI de oxitocina profiláctica en la tercera etapa del parto. Además, la mayoría de las mujeres que dan luz en el hospital participante tiene una línea IV en sitio. El objetivo del estudio será evaluar el efecto de la vía de administración de 10 UI de oxitocina en la pérdida promedio de sangre. Las mujeres elegibles para participar serán aquéllas que proporcionen su consentimiento informado y tengan partos vaginales. Las participantes se aleatorizarán para recibir:

- 10 UI de oxitocina mediante infusión IV

o

- 10 UI de oxitocina mediante inyección IM

De acuerdo con el esquema de aleatorización, se administrarán 10 UI de oxitocina tan pronto como sea posible, después del nacimiento del bebé. La infusión IV se realizará colocando 10 UI de oxitocina en un frasco de solución salina de 500 cc y el tiempo en que debe realizarse la perfusión será dentro de 40 minutos (por Ej., 12cc/min o 240ggts/min). Todas las participantes del estudio recibirán la oxitocina por infusión IV o por la inyección IM, basado en la asignación aleatorizada, y el placebo correspondiente mediante la vía no-asignada.

Para evitar cualquier prejuicio potencial que el conocimiento de la vía de administración de oxitocina puede tener en la cuantificación de la pérdida de sangre, los miembros del personal del estudio y los participantes no conocerán la asignación de la vía de administración del fármaco activo. La pérdida de sangre se medirá en un recipiente calibrado durante una hora. Si una mujer experimenta sangrado excesivo o HPP, se le administrará el tratamiento que dicte la norma de atención del centro de estudio.

Independientemente del diagnóstico de HPP, a las mujeres que tengan parto vaginal y que otorguen su consentimiento se les medirá la pérdida de sangre después del parto con un recipiente estandarizado y mediante el uso de otros indicadores y resultados registrados durante el período posparto inmediato, a fin de evaluar la relación entre el índice de shock y los resultados de la HPP. La pérdida de sangre se medirá durante una hora y de diagnosticarse HPP, se seguirá midiendo hasta que cese el sangrado activo. Se tomará la frecuencia cardiaca y la presión arterial en intervalos definidos (15, 30, 45 y 60 minutos) para documentar los cambios hemodinámicos que ocurran en el posparto inmediato.

***Criterios de elegibilidad***

*Criterios de inclusión*

Todas las mujeres que se presenten a un hospital participante con trabajo de parto activo para dar a luz a un feto vivo serán potencialmente elegibles para su enrolamiento en el estudio. Se enrolarán en el estudio mujeres que se presenten para recibir atención por parto vaginal. Independientemente de que se les diagnostique HPP, en mujeres que proporcionen su consentimiento y que tengan parto vaginal se recolectará y medirá la pérdida de sangre en el posparto, así como otros indicadores y resultados registrados en el posparto inmediato a fin de evaluar la relación existente entre el índice de shock y los resultados de la HPP.

*Criterios de exclusión*

Se excluirá a las mujeres:

- Programadas o transferidas para cesárea
- Que rechazan que se les coloque una via intravenosa durante el parto ( venoclisis)
- Que no pueden otorgar consentimiento informado por presentar disminución en sus facultades mentales, angustia durante el parto o por otras razones
- Que no se muestran dispuestas y/o no pueden responder el cuestionario sobre antecedentes

Si en cualquier momento del estudio la mujer desea retirarse, se le dejará de considerar como participante y no se le dará seguimiento como tal.

***Consentimiento informado y procedimientos previos al parto***

El consentimiento informado lo obtendrá personal capacitado del estudio una vez que las mujeres hayan sido ingresadas al hospital y a su llegada a la sala de trabajo de parto. Si en la opinión del personal de la institución la mujer se encuentra en una etapa demasiado avanzada del parto como para otorgar su consentimiento informado de manera adecuada, no se le considerará elegible para enrolarla en el estudio. Las mujeres que se nieguen a participar recibirán toda la atención médica necesaria de conformidad con la práctica estándar del hospital. A las mujeres que deseen participar se les pedirá que lean y firmen el consentimiento informado; a quienes no sepan leer, se les leerá y explicará el formulario e indicarán su consentimiento con una marca, como por ejemplo la huella digital de su pulgar. Si en cualquier momento del estudio la mujer indica que no desea continuar, no se la incluirá en la recolección de datos restante.

Los antecedentes y los datos iniciales se obtendrán y documentarán en el Formulario 1. El nivel de hemoglobina previo al parto de todas las participantes se medirá y registrará con el hemoglobinómetro Hemocue® + micro cubetas (HemoCue, Ängelholm, Suecia). También se registrarán las medidas iniciales de frecuencia cardiaca y presión arterial al momento de inscribirse en el estudio.

***Aleatorización y reclutamiento***

Se aleatorizará a todas las participantes para que reciban manejo profiláctico con oxitocina en la tercera etapa del parto mediante infusión IV o inyección IM; la asignación se encontrará en paquetes del estudio numerados consecutivamente. Durante la segunda etapa, antes del parto de cada mujer, el personal del estudio abrirá el siguiente paquete consecutivo de aleatorización. Ya que el estudio será doble-ciego y controlado con placebo, cada paquete contendrá una ampolla de 10 UI de oxitocina y otra de placebo semejante a la oxitocina de 10 UI de solución salina. Cada ampolla tendrá una identificación que indicará la vía asignada de administración del contenido.

Las ampollas de 10 UI de oxitocina y las ampollas de placebo semejante (que contendrán 10 UI de solución salina) serán preparadas por un laboratorio/farmacéutico local. Las ampollas preparadas para el estudio (e.g. paquetes del estudio) tendrán que ser almacenados en un refrigerador para mantener la cadena de frio necesaria para la oxitocina hasta el momento de la administración a las madres. El código de aleatorización lo generará anticipadamente el personal de Gynuity Health Projects en Nueva York. La asignación aleatoria se efectuará en bloques de 10 y se generará con un programa de computadora. Sólo la coordinadora del estudio, que pertenece a Gynuity tendrá acceso al esquema de aleatorización. Una vez asignada a un grupo de estudio, se considerará a la mujer como participante enrolada en el mismo.

El personal del estudio observará a las participantes y documentará toda intervención realizada durante el parto, incluido cualquier otro componente del MATEP y la colocación de la línea IV (de no haberse colocado todavía). Asimismo, el personal anotará si el parto fue inducido o estimulado y de ser posible, el medicamento/método utilizado para ello y la hora de administración del procedimiento. Si se estimula el parto con oxitocina, se deberá anotar que se interrumpió el uso del agente al salir la cabeza del feto.

***Intervención***

De acuerdo con el esquema de aleatorización, se administrarán 10 UI de oxitocina mediante infusión IV o por vía IM tan pronto como sea posible, y el placebo por la vía no-asignada después del nacimiento del bebé.

Inmediatamente después del nacimiento del bebe, se medirá la pérdida de sangre durante una hora con un recipiente calibrado. También se anotará la cantidad total de sangre perdida cuando se detenga la hemorragia activa. De presentarse sangrado excesivo o HPP que requiera tratamiento, se documentará la pérdida de sangre también al momento del diagnóstico. Se espera que en el parto algo de sangre salpique la ropa quirúrgica y las batas del personal clínico presente y por ello, se debe hacer todo lo posible por minimizar ese tipo de pérdida. Asimismo, se debe recordar que los intersticios placentarios contienen sangre materna (un 9% del peso placentario). Consideramos que la sobreestimación (líquido amniótico) y la subestimación (pérdida de sangre) tenderán a distribuirse entre los dos grupos de estudio, por lo tanto no se necesitará corregir la medición de sangre perdida ocasionada por esas inexactitudes.

Si una mujer experimenta sangrado excesivo o HPP y requiere tratamiento, se la deberá asistir de acuerdo a las normas de atención del hospital participante. De diagnosticarse HPP, la medición de la cantidad de sangre perdida continuará hasta que cese la hemorragia. Los proveedores de salud podrán diagnosticar la HPP en cualquier momento, pero si la pérdida de sangre alcanza los 500 ml de acuerdo a las marcas del recipiente calibrado, se deberá diagnosticar HPP e iniciar el tratamiento estándar del hospital.

El personal del estudio mantendrá en observación a las participantes y registrará todas las intervenciones efectuadas durante el parto, incluida la administración de oxitocina profiláctica del estudio. Asimismo, anotará si el parto fue inducido o estimulado antes del alumbramiento y de ser posible, el medicamento proporcionado, y la hora y dosis en que se administró. Se pedirá a la proveedora o proveedor que monitoree a la mujer durante una hora después del nacimiento del bebé para detectar efectos secundarios. Las participantes también responderán preguntas sobre los efectos secundarios antes del alta hospitalaria.

A fin de documentar los cambios en los valores del índice de shock durante la primera hora después del parto, se registrarán la frecuencia cardiaca y la presión arterial de todas las mujeres enroladas a intervalos de 15, 30, 45 y 60 minutos. Se tomarán dichas mediciones independientemente del diagnóstico de HPP y se anotará la hora exacta de cada medición. Se proporcionarán dispositivos automáticos a todos los centros de estudio para medir la presión arterial y la frecuencia cardiaca a fin de estandarizar dichas mediciones.

Se medirá la hemoglobina posparto con el hemoglobinómetro Hemocue® + micro cubetas por lo menos 24 horas después del nacimiento y antes de que la mujer sea dada de alta del hospital. Si se le han administrado líquidos IV, se medirá la hemoglobina por lo menos 12 horas después de la remoción de la línea IV y antes del alta.

***Formularios de documentación del estudio***

*Admisión*. El formulario documentará las características demográficas básicas y el historial médico y obstétrico para determinar la elegibilidad de las mujeres para participar en el estudio. También se registrará la firma del consentimiento informado. En el formulario de admisión se asentarán las mediciones iniciales de Hb, frecuencia cardiaca y presión arterial. El formulario de admisión sólo se llenará para las mujeres que hayan otorgado su consentimiento.

*Aleatorización/Parto*. El formulario documentará el número de aleatorización e información relevante obtenida durante y después del parto e incluirá la hora del nacimiento y los eventos o intervenciones realizadas durante la tercera etapa del parto, en especial en lo relacionado a la administración de agentes uterotónicos y otros componentes del manejo activo. La presión arterial y la frecuencia cardiaca se medirán a los 15, 30, 45 y 60 minutos después del nacimiento del bebé y se asentarán en este formulario. También se registrarán a intervalos de 15 minutos las lecturas de pérdida de sangre que se realicen durante la primera hora después del parto. Asimismo, se anotará toda información relacionada con el diagnóstico de la HPP. El formulario se llenará para todas las mujeres que hayan otorgado su consentimiento.

*Seguimiento.* El formulario se utilizará para anotar información sobre efectos secundarios, eventos adversos o problemas que se presenten en el seguimiento después del parto y antes del alta hospitalaria. En ese punto se medirá y anotará el nivel de hemoglobina posparto. El formulario se llenará para todas las mujeres que hayan otorgado su consentimiento.

*Formulario de eventos adversos serios (EAS)*. Se utilizará para documentar cualquier EAS conforme a las definiciones presentadas a continuación.

Se define como evento adverso serio al que produce:

- Muerte de la mujer
- Hospitalización prolongada (o una nueva internación);
- Nueva discapacidad o incapacidad significativa y/o permanente;
- Amenazas a la vida de la paciente; o
- Anomalía congénita.

El investigador deberá reportar todo EAS dentro de los primeros 3 a 5 días a partir de haberse enterado de ellos. El informe se hará por fax o escaneada a la atención de SAE DESK al número **+1-212-448-1260** o al correo electrónico **iplatais@gynuity.org**. Cabe hacer notar que los decesos maternos se deberán informar dentro de las primeras 24 horas de haber ocurrido. El informe deberá ser acompañado por una descripción detallada por escrito que contenga los datos de la paciente, la descripción del evento o problema, resultados de laboratorio relevantes e información relacionada con trastornos preexistentes. Como se describió con anterioridad, el hospital recibirá una dotación de formularios para EAS que se deberán llenar según se requiera.

Al reportar un EAS a la coordinación del estudio, el investigador *in situ* deberá proteger la confidencialidad de la paciente, por lo que no mencionará nombres ni direcciones. El informe contendrá solamente el código único de la participante y el investigador deberá conservar dicho código para facilitarle al coordinador del estudio o a las autoridades de reglamentación de medicamentos la verificación de datos.

**Manejo y monitoreo de los datos**

Se enviarán copias de todos los formularios de las primeras cinco mujeres enroladas a la coordinadora del estudio a Gynuity Health Projects para revisarlos antes de proseguir con el reclutamiento. Posteriormente, se enviarán periódicamente copias de los formularios a Gynuity Health Projects a Nueva York. Se conservarán en cada hospital participante todos los formularios originales.

Se capturará la información en una base de datos para revisarla posteriormente a fin de detectar inconsistencias en la lógica de la base y para depurarla y analizarla. Se mantendrá el doble ciego para la duración del estudio hasta que todos los datos de los formularios estén capturados en la base de datos y verificados.

***Monitoreo de datos y seguridad***

Gynuity Health Projects monitoreará el avance de cada hospital participante. Personal de monitoreo capacitado visitará los hospitales por lo menos dos veces a lo largo del estudio para observar las prácticas y revisar la documentación a fin de verificar que:

- Se protejan los derechos y el bienestar de los sujetos humanos;
- Los datos se recopilen de acuerdo con el protocolo, sean precisos, estén completos y sean de la más alta calidad e integridad;
- El ensayo cumpla con el protocolo aprobado y Buenas Prácticas Clínicas.

El personal de monitoreo prestará especial atención a que se garantice que las mujeres enroladas en el estudio cumplan con los criterios de elegibilidad, que el reclutamiento avance adecuadamente, el consentimiento informado se documente de forma apropiada, las y los proveedores observen el esquema de aleatorización, los datos se reporten correctamente y se eviten violaciones/desviaciones del protocolo. Cualquier desviación del protocolo o de las prácticas que detecte el personal de monitoreo se analizará con las y los investigadores del centro de estudio y se tomarán medidas correctivas.

***Análisis y divulgación de resultados***

Se realizarán análisis bivariados, estratificados por grupo de estudio. Se efectuará una regresión logística no ajustada para variables categóricas y una regresión logística ajustada para variables dependientes continuas.

Para la comparación de los dos grupos de estudio (oxitocina IM versus infusión de oxitocina IV), los análisis principales incluirán:

- Pérdida promedio de sangre en el posparto (resultado primario)
- % de mujeres con pérdida de sangre ≥500 ml (resultado primario
- Mediana de la pérdida de sangre en el posparto y rango intercuartílico
- % de mujeres con pérdida de sangre ≥1000 ml
- Cambio promedio en Hb medida antes y después del parto
- % de mujeres con disminución de hemoglobina posparto de 2 g/dl o mayor
- % de mujeres que presentan una tercera etapa del parto prolongada
- % de mujeres que recibió oxitócicos adicionales u otras intervenciones para manejar el sangrado
- % de mujeres que experimentaron efectos secundarios

Se registrará el uso de cualquier otro componente del MATEP, tal como la tracción controlada del cordón, el masaje uterino, etc., que se empleen en los hospitales participantes.

Para evaluar la relación entre índice de shock y severidad del sangrado, correlacionaremos los valores del índice de shock, la frecuencia cardiaca y las mediciones de presión arterial con los niveles de pérdida de sangre que se registrarán a los 15, 30, 45 y 60 minutos después del parto. Se analizarán los valores absolutos y los cambios medidos en dichos valores durante la primera hora después del parto. Asimismo, exploraremos las asociaciones entre esos signos/síntomas clínicos y otros resultados de HPP, incluida la provisión de intervenciones adicionales para controlar el sangrado, transfusiones y otros resultados severos.

Los datos serán propiedad de Gynuity Health Projects. Las y los investigadores principales y la coordinación del estudio trabajarán conjuntamente para analizar los resultados y preparar al menos un manuscrito para someterlo a una o más revistas científicas arbitradas. Asimismo, los miembros del equipo del estudio presentarán los resultados en conferencias y reuniones, como se considere conveniente. Se listarán los nombres de las y los investigadores principales y del personal clave de Gynuity como autores de cualquier publicación o presentación que describa los resultados del estudio.

**Sujetos humanos**

El estudio se realizará de conformidad con la versión actual de la Declaración de Helsinki. El protocolo requiere que un consentimiento informado se encuentre a disposición de todas las participantes. Un Consejo de Revisión Institucional/Comité de Ética debe aprobar el protocolo antes de reclutar a la primera participante del estudio.

A todos los documentos y registros de las participantes del estudio se les asignarán identificadores únicos que se crearán con las iniciales de las mismas y un código numérico. No se asociarán los nombres de las mujeres a los documentos del estudio para preservar su confidencialidad. Todo registro se conservará en archiveros cerrados con llave y sólo personal autorizado del estudio tendrá acceso a ellos. No se divulgará ningún dato clínico sin la autorización de la participante excepto en los casos en que fuera necesario para fines de monitoreo.

**Referencias**

1. WHO, *WHO recommendations for the prevention of postpartum haemorrhage.* WHO, 2007.

2. Midwives;, I.C.o. and I.F.o.G.a. Obstetrics, *Prevention and Treatment of Post-partum haemorrhage: New Advances for Low Resource Settings.* 2006: p. 1-4.

3. Cotter, A.M., Ness Amen, and J.E. Tolosa, *Prophylactic oxytocin for the third stage of labour.* Cochrane Database Systems Review; John Wiley & Sons, Ltd., 2001(4).

4. Prendiville, W.J., et al., *The Bristol third stage trial: active versus physiological management of third stage of labour.* BMJ, 1988. **297**(6659): p. 1295-300.

5. de Groot, A.N.J.A., et al., *A placebo-controlled trial of oral ergometrine to reduce postpartum hemorrhage.* Acta Obstetricia et Gynecologica Scandinavica, 1996. **75**(5): p. 464 - 468.

6. Nordstrom, L., et al., *Routine oxytocin in the third stage of labour: a placebo controlled randomised trial.* British Journal of Obstetrics and Gynaecology, 1997. **104**(7): p. 781-6.

7. Pierre, F., L. Mesnard, and G. Body, *For a systematic policy of i.v. oxytocin inducted placenta deliveries in a unit where a fairly active management of third stage of labour is yet applied: results of a controlled trial.* European Journal of Obstetrics, Gynecology, and Reproductive Biology, 1992. **43**(2): p. 131-5.

8. Choy, C.M., et al., *A randomised controlled trial of intramuscular syntometrine and intravenous oxytocin in the management of the third stage of labour.* International Journal of Gynecology and Obstetrics, 2002. **109**(2): p. 173-7.

9. Rashid, M., A. Clark, and M.H. Rashid, *A randomised controlled trial comparing the efficacy of intramuscular syntometrine and intravenous syntocinon, in preventing postpartum haemorrhage.* J Obstet Gynaecol, 2009. **29**(5): p. 396-401.

10. Ilancheran, A. and S.S. Ratnam, *Effect of oxytocics on prostaglandin levels in the third stage of labour.* Gynecol Obstet Invest, 1990. **29**(3): p. 177-80.

11. Fugo, N.W. and W.J. Dieckmann, *A comparison of oxytocic drugs in the management of the placental stage.* Am J Obstet Gynecol, 1958. **76**(1): p. 141-6.

12. Sorbe, B., *Active pharmacologic management of the third stage of labor. A comparison of oxytocin and ergometrine.* Obstet Gynecol, 1978. **52**(6): p. 694-7.

13. Howard, W.F., P.R. McFadden, and W.C. Keettel, *Oxytocic Drugs in Fourth Stage of Labor.* JAMA, 1964. **189**: p. 411-3.

14. Tsu, V.D., et al., *Reducing postpartum hemorrhage in Vietnam: assessing the effectiveness of active management of third-stage labor.* J Obstet Gynaecol Res, 2006. **32**(5): p. 489-96.

15. Poeschmann, R.P., W.H. Doesburg, and T.K. Eskes, *A randomized comparison of oxytocin, sulprostone and placebo in the management of the third stage of labour.* Br J Obstet Gynaecol, 1991. **98**(6): p. 528-30.

16. Newton, M., et al., *Blood loss during and immediately after delivery.* Obstet Gynecol, 1961. **17**: p. 9-18.

17. Gulmezoglu, A.M., et al., *WHO multicentre randomised trial of misoprostol in the management of the third stage of labour.* Lancet, 2001. **358**(9283): p. 689-95.

18. Gibbens, D., et al., *The circulating levels of oxytocin following intravenous and intramuscular administration of Syntometrine.* J Obstet Gynaecol Br Commonw, 1972. **79**(7): p. 644-6.

19. Lyndon A, L.D., Shields L, Melsop K, Bingham B, Main E (Eds). *Improving Health Care Response*

*to Obstetric Hemorrhage. (California Maternal Quality Care Collaborative Toolkit to Transform Maternity*

*Care)*, in *Improving Health Care Response to*

*Obstetric Hemorrhage*. 2010, California Maternal Quality Care Collaborative,.

20. Crall, H.D. and D.R. Mattison, *Oxytocin pharmacodynamics: effect of long infusions on uterine activity.* Gynecol Obstet Invest, 1991. **31**(1): p. 17-22.

21. Cunningham, F.G. and J.W. Williams, *Williams obstetrics*. 21st ed. 2001, New York: McGraw-Hill. x, 1668 p.

22. Gonser, M. and P. Kahle, *Estimated linear relationship between ductus venosus index and gestational age.* Ultrasound Obstet Gynecol, 1994. **4**(1): p. 85; author reply 86.

23. Prendiville, W.O.C., M., *Active Management of the Third Stage of Labor*. 1 ed. A Textbook of Postpartum Hemorrhage, ed. C.B.-L.L.G.K.A.B.L.M. Karoshi. 2006, Duncow: Sapiens Publishing. 462.

24. Seitchik, J., et al., *Oxytocin augmentation of dysfunctional labor. IV. Oxytocin pharmacokinetics.* Am J Obstet Gynecol, 1984. **150**(3): p. 225-8.

25. Gonser, M., *Labor induction and augmentation with oxytocin: pharmacokinetic considerations.* Arch Gynecol Obstet, 1995. **256**(2): p. 63-6.

26. Huh, W.K., D. Chelmow, and F.D. Malone, *A Double-Blinded, Randomized Controlled Trial of Oxytocin at the Beginning versus the End of the Third Stage of Labor for Prevention of Postpartum Hemorrhage.* Gynecologic and Obstetric Investigation, 2004. **58**(2): p. 72-6.

27. Soriano, D., et al., *A prospective cohort study of oxytocin plus ergometrine compared with oxytocin alone for prevention of postpartum haemorrhage.* Br J Obstet Gynaecol, 1996. **103**(11): p. 1068-73.

28. Villar, J., et al., *Systematic review of randomized controlled trials of misoprostol to prevent postpartum hemorrhage.* Obstetrics and Gynecology, 2002. **100**(6): p. 1301-12.

29. Sheldon, W.R.D., Jill; Winikoff, Beverly; Blum, Jennifer; Trussell, James, *How effective are the components of active management of the third stage of labor?* BMC Pregnancy and Childbirth, 2013. **13**(46).

30. Davies, G.A., et al., *Maternal hemodynamics after oxytocin bolus compared with infusion in the third stage of labor: a randomized controlled trial.* Obstet Gynecol, 2005. **105**(2): p. 294-9.

31. SICOR Pharmaceuticals, I., *Oxytocin Injection, USP Synthetic 10 units/1 mL*, FDA, Editor. 2008: Irvine.

32. MN, S., *Measurement of blood loss: review of the literature.* J Midwifery Wom Heal, 2010. **55**(1): p. 20-27.

33. Bonnar, J., *Massive obstetric haemorrhage.* Baillieres Best Pract Res Clin Obstet Gynaecol, 2000. **14**(1): p. 1-18.

34. Pacagnella, R.C., et al., *A systematic review of the relationship between blood loss and clinical signs.* PLoS One, 2013. **8**(3): p. e57594.

35. Le Bas, A., et al., *Use of the "obstetric shock index" as an adjunct in identifying significant blood loss in patients with massive postpartum hemorrhage.* Int J Gynaecol Obstet, 2014. **124**(3): p. 153-5.

36. Nathan, H., et al., *Shock index: an effective predictor of outcome in postpartum haemorrhage?* BJOG, 2015. **122**(2): p. 268-75.

37. El Ayadi, A.M., et al., *Vital Sign Prediction of Adverse Maternal Outcomes in Women with Hypovolemic Shock: The Role of Shock Index.* PLoS One, 2016. **11**(2): p. e0148729.

38. Khan, G.Q., et al., *Controlled cord traction versus minimal intervention techniques in delivery of the placenta: a randomized controlled trial.* Am J Obstet Gynecol, 1997. **177**(4): p. 770-4.

39. Derman, R.J., et al., *Oral Misoprostol in preventing postpartum haemorrhage in resource-poor communities: a randomised controlled trial.* The Lancet, 2006. **368**(9543): p. 1248-53.

40. Dzuba, I., *Reduciendo el sangrado postparto: Uterotónicos profilácticos y consideraciones para su uso* in *XXI Congreso FLASOG*. 2014: Guayaquil, Ecuador.

41. Anger, H., *Route of Administration of Oxytocin in Prevention of Postpartum Hemorrhage*, in *XXI FIGO World Congress of Gynecology and Obstetrics*. 2015: Vancouver, Canada.

**Apéndice. Información para la participante y consentimiento informado**

**La administración de oxitocina intravenosa versus intramuscular y su relación con el sangrado posparto y otros signos clínicos: un estudio aleatorizado y controlado por placebo**

Para ser leído a cada posible participante en su propio idioma. Una copia del consentimiento firmado se le entregará a la participante.

Hoja de información para las mujeres que durante el trabajo de parto asisten a un hospital que participa en el proyecto de investigación: “**Administración de oxitocina intravenosa e intramuscular en la tercera etapa del parto para la prevención de la hemorragia posparto”**

Nombre del Investigador Principal:

Nombre de la Institución:

Estoy trabajando para el Hospital………………………….: Estamos estudiando las maneras de reducir la pérdida de sangre importante que ocurre en algunas mujeres después del parto.

**Finalidad:** Estamos realizando un estudio para comparar dos formas de administrar una medicina llamada oxitocina y ver si tienen efectos diferentes en el sangrado posparto. La oxitocina se administra en este hospital a todas las mujeres después del nacimiento del bebé para evitar que sangren de manera excesiva, y es el procedimiento habitual en hospitales de todo el mundo. Un método de administrar la oxitocina es aplicar una inyección intramuscular y otro es con una aguja a través de una vena de la mano o del brazo. Los dos métodos de administración de oxitocina son eficaces para disminuir el sangrado después del parto. El estudio se está realizando en el Hospital Vidal, donde participarán un total de 486 mujeres.

Si está de acuerdo en participar en el estudio, se le administrará la medicina del estudio que es oxitocina y un placebo – uno por inyección intramuscular y otro intravenoso inmediatamente después de que haya nacido su bebé. Tendrá las mismas probabilidades de recibir la oxitocina por cualquiera de las dos vías (intramuscular o intravenosa); pero ni usted ni el médico sabrán por cuál de las dos vías le estarán administrando la oxitocina

**Procedimiento:** Si usted decide participar en el estudio, le haremos preguntas sobre sus antecedentes médicos antes del parto y le haremos algunas preguntas personales. Antes del parto, le pincharemos el dedo para tomarle una muestra de sangre. Después de que nazca su bebé, utilizaremos una bolsa plástica para recolectar y medir la sangre que pierda para conocer la cantidad de sangre perdida y si es necesario algún tratamiento. Asimismo, le tomaremos la presión arterial y el pulso cada 15 minutos durante la primera hora después del parto para controlar su estado de salud.

**Duración:** Su participación en el estudio durará el tiempo que Ud. se encuentre internada. De tener una hemorragia posparto se le medirá la pérdida de sangre durante un mínimo de 60 minutos y se proseguirá hasta el cese del sangrado; también se le medirán los niveles de glóbulos rojos 24-48 horas después del parto.

**Beneficios:** Si usted acepta participar en este estudio, puede que obtenga o no beneficios médicos directos. Como le tomaremos sangre, podremos darle información si tiene anemia. También vigilaremos de cerca la cantidad de sangre que pierda después del parto. Esperamos que la información que se obtenga como resultado de este estudio, beneficie en el futuro a otras mujeres. Con los resultados del estudio, podríamos saber si una forma de administrar la oxitocina (por inyección muscular o intravenosa) es mejor que la otra.

Los riesgos de participar en el estudio serán la posible molestia del pinchazo en el dedo para sacar la muestra de sangre, la pequeña posibilidad de que le quede un moretón y la todavía más lejana probabilidad de que se infecte el lugar de donde le tomen la muestra. Como mencionamos anteriormente, podría experimentar también una ligera molestia por la aguja usada para administrarle la medicina (oxitocina y/o placebo); pero incluso si decide no participar en el estudio, es posible que de todas maneras reciba la misma medicación.

**Compensación:** Usted no recibirá ninguna remuneración por participar en el estudio. Cualquier tipo de complicación derivada de su participación del estudio será atendida de acuerdo a los protocolos de tratamiento de este hospital, sin costo alguno para usted.

**Costos:** La participación en este estudio no tiene costo, ni le ocasionará gastos adicionales.

**Confidencialidad:** Cualquier información que usted brinde durante el estudio será confidencial. Su nombre completo no aparecerá en ningún documento del estudio y sólo el personal que participe en este estudio tendrá acceso a la información que usted brinde o que se obtenga de su historia clínica hospitalaria. Le brindaremos información actualizada acerca de éste u otros estudios que puedan afectar su salud o bienestar.

**Derecho a negarse a participar o abandonar:** La participación en el estudio es voluntaria. Usted tiene la libertad de elegir si desea o no participar en el estudio. También tiene la libertad de abandonar el estudio en cualquier momento si así lo desea, sin tener que dar explicaciones sobre su decisión. El retirarse del estudio no le representará ninguna penalidad o pérdida de beneficios a los que tiene derecho.

**Alternativas a la participación:** Si por cualquier motivo usted no es elegible para el estudio, o decide no participar, igual recibirá los tratamientos adecuados y los medicamentos necesarios antes, durante y después del parto.

**Con quién contactarse:** Si tiene alguna pregunta puede hacerla ahora o en el momento del parto. Si desea hacer preguntas en otro momento, puede contactarse con el medico investigador o con el Dr. Guillermo Carroli en el Centro Rosarino de Estudios Perinatales. Moreno 878. Piso 6. 2000. Rosario. TE: 0341 448-3887.

Esta propuesta ha sido revisada y aprobada por el Comité de Etica del Centro Rosarino de Estudios Perinatales, que es un comité cuya tarea es asegurar que los participantes estén protegidos de cualquier daño. Si desea saber más sobre el Comité de Etica, contáctese con: Dr. Rafael Pineda, TE: 0341 425-9245

¿Tiene preguntas acerca del estudio?

¿Está dispuesta a participar?

**La administración de oxitocina intravenosa versus intramuscular y su relación con el sangrado posparto y otros signos clínicos: un estudio aleatorizado y controlado por placebo**

Nombre de la mujer:……………………………………………………………………………………………

He leído o me han leído la información relacionada con el estudio y entiendo qué se espera de mí en caso de que decida participar. Han respondido y aclarado a satisfacción todas mis preguntas y preocupaciones. Entiendo que puedo salir del estudio en el momento que lo desee sin explicar la razón y que esa decisión no impedirá que reciba los servicios de salud normales que se ofrecen en este hospital.

Estoy de acuerdo en participar en el estudio.

Firma/huella del pulgar de la mujer …………………………………………………….

Fecha……………………………………….

Firma del miembro del personal del estudio………………………………………………

**En caso de tener preguntas relacionadas con su participación en el estudio, favor de comunicarse con:**

**[Insertar nombre y teléfono de la persona de contacto del estudio / Investigador(a) Principal]**
