# Supplementary material for: Does route matter? Impact of route of oxytocin administration on postpartum bleeding: A double-blind, randomized controlled trial
Source: PLoS One. 2019 Oct 1;14(10):e0222981. doi: 10.1371/journal.pone.0222981 (PMC6772050; doi:10.1371/journal.pone.0222981)
Supplement: S2 Protocol — (DOC) [file pone.0222981.s005.doc]

**Protocol#**: 3008

**Protocol Title:** The administration of intravenous oxytocin versus its intramuscular administration in the third stage of labor and its relationship with postpartum bleeding and other clinical signs: a double-blinded, placebo-controlled, randomized trial

**Princicpal investigator**

Dra. Beverly Winikoff, Gynuity Health Projects, Estados Unidos

**Co-investigators:**

# Jill Durocher, Gynuity Health Projects, USA

# Ilana Dzuba, Gynuity Health Projects, USA

# Dr. Guillermo Carroli, Centro Rosarino de Estudios Perinatales (CREP), Argentina

# Co-investigators from study location: *Hospital J.R. Vidal, Corrientes, Argentina*

# Prepared by:

# Gynuity Health Projects

# 15 East 26^th^ Street, Suite 801

# NY, NY 10010

**Background**

Postpartum hemorrhage is a major cause of maternal death worldwide and is most commonly a result of uterine atony, the failure of the uterus to contract to stem blood loss following delivery. Active management of the third stage of labor (AMTSL) is recommended in high- and low-resource settings as an effective method to prevent postpartum hemorrhage secondary to uterine atony. The administration of a synthetic uterotonic stimulates the smooth muscles of the uterus to increase tone and is a major component of AMTSL that is practiced in many parts of the world. Oxytocin is universally considered the uterotonic of choice for this indication. Comparative studies of oxytocin either in combination with other components of active management or alone show that it reduces the risk of bleeding and PPH.

The published literature on the use of oxytocin to prevent PPH is relatively large, but it describes many variations in its administration (e.g. route, dose and timing). For example, in some studies oxytocin is administered intravenously (IV) ; and in others it is administered intramuscularly (IM) We are not aware of any published studies that directly compare IV and IM administration of oxytocin during the third stage of labor and the ongoing underlying assumption seems to be that the route of administration has no effect on postpartum blood loss . However, results from a 1972 study on circulating oxytocin levels after IV, IM and subcutaneous injections of Syntometrine (5 IU oxytocin and 0.5mg ergometrine) in a small sample of women in the third stage of labor (n=26) indicated that plasma oxytocin levels rise more rapidly following IV and reach a much higher peak than after IM .

These variations in route of oxytocin administration noted in the literature are also reflected in clinical practice and guidelines, and there does not seem to be agreement or a universal standard procedure for providing prophylactic oxytocin in the third stage of labor. Some guidelines specify IM administration, which is the most reasonable route for lower-levels of care and other settings where IV insertion is not feasible because of required skills/permission and because it complicates mobility and care of patients . Other guidelines either do not specify one route over another or they offer the two as equal alternatives .

It is likely that much of the variation in clinical protocols on oxytocin administration can be attributed to inadequate or disputed data on the pharmacokinetics and pharmacodynamics of oxytocin.The time to steady state plasma concentration depends on the half life of a drug. The longer the half life, the longer it takes to achieve a steady state. The half life of oxytocin is usually reported as approximately three minutes, although some articles report half-lives up to 20 minutes. Regardless of route of administration, oxytocin is fast-acting. Uterine sensitivity to oxytocin increases throughout pregnancy due to higher concentration of oxytocin receptors. After IV injection, the onset of action is almost immediate, within one minute, and a slightly slower 2-4 minutes after IM injection . With IV infusion, the uterine response is gradual and achieves steady-state plasma concentrations after 15-40 minutes ; sustained contractions, although speed of delivery characterized by needle gauge, catheter size and quantity of fluid for dilution, can affect time to steady state.

While a comparison of IV and IM administration of oxytocin during the third stage of labor has not been rigorously explored, authors of several papers question a potential effect of the route of administration, particularly in relation to speed of delivery and its effect on preventing postpartum blood loss. An unpublished secondary analysis from a large clinical trial in which women received oxytocin prophylaxis in the third stage of labor suggests that route of administration matters, particularly when oxytocin is the only AMTSL component provide. Mean postpartum blood loss was 336 ml among women who received only oxytocin IM prophylaxis in the third stage of labor (n=2845) and was 60 ml lower (277 ml) among women who received only oxytocin IV prophylaxis (n=785). The women who received oxytocin prophylaxis via IV had a reduced risk of postpartum blood loss ≥700ml compared to women who received oxytocin IM (OR 0.24, 95% CI 0.12-0.51). The probability of such blood loss was 0.5% instead of 2.1%. Of the 785 women who received oxytocin by IV, the vast majority of women receiving oxytocin IV were given bolus injections. Forthcoming results from a WHO-sponsored study on the components of AMTSL will include a comparison of the effect of IV and IM oxytocin on PPH. Another study found that IV bolus of oxytocin resulted in a statistically significant lower estimated mean blood loss as compared to IV infusion of oxytocin (358 ml vs 424 ml, p=0.029). In this study, blood was not measured in a calibrated container and blood loss was estimated by clinical providers’ visual assessments, however it does raise the question of whether IV bolus and IV infusion are as similar as they are assumed to be.

Oxytocin is widely considered to be a safe drug when used prophylactically to prevent PPH. Side effects are uncommon in women who deliver vaginally, however nausea and vomiting have been reported . A study comparing maternal hemodynamics after oxytocin bolus or infusion in the third stage of labor following vaginal delivery showed that bolus administration of oxytocin 10 IU is not associated with adverse hemodynamic responses and can safely be administered to women with intravenous access in the third stage of labor for postpartum hemorrhage prophylaxis.

En el presente protocolo, describimos un estudio controlado y aleatorizado que busca evaluar el efecto de la vía de administración de 10 UI de oxitocina en la pérdida promedio de sangre en hospitales de nivel terciario, donde la política de atención estándar es la administración de oxitocina profiláctica en la tercera etapa del parto. (La dosis de oxitocina profiláctica varía de 5 a 40 UI, pero dado que el uso de 10 UI es más común en varios modos de administración y es el recomendado por la Organización Mundial de la Salud, estandarizaremos una dosis de 10 UI para todos los grupos de tratamiento.) En nuestro análisis, se documentará y controlará la práctica de otros componentes del MATEP. También evaluaremos los efectos secundarios y eventos adversos asociados a cada vía de administración, incluida la pérdida de sangre.

In this protocol, we describe a randomized controlled study design that seek to evaluate the effect of route of administration of 10 IU oxytocin on mean blood loss at a tertiary-level hospital where institutional policy requires prophylactic oxytocin in the third stage of labor. (The dose for oxytocin prophylaxis can range from 5 to 40 IU, but the use of 10 IU is more common across different modes of administration and is recommended by the World Health Organization , so we will standardize a dose of 10 IU in all treatment groups.) The practice of other AMTSL components will be documented and controlled for in our analysis. We will also evaluate the side effects and adverse events associated with each route of administration, including blood loss.

The results of these studies will fill a void in the published literature about whether route of oxytocin administration during the third stage of labor has an effect on blood loss. Furthermore, the results of this study could have important implications for use of non-parenteral uterotonics as prophylaxis in the third stage of labor, such as misoprostol, an E1 prostaglandin analog that is effective in reducing acute and severe PPH. Current guidelines recommend that oxytocin delivered by IV infusion or IM injection is preferred over misoprostol, however, a difference in effectiveness of route of oxytocin administration to stem postpartum bleeding favoring IV would suggest a greater role for misoprostol where oxytocin is available but IV administration is not feasible.

Complementary to ongoing research on treatment of PPH, there is a need for evaluation of new clinical indicators that could facilitate early diagnosis and treatment of PPH. For now, timely and appropriate clinical intervention largely depends on providers’ ability to assess blood loss visually to determine whether treatment might be required. Estimating postpartum blood loss repeatedly has been shown to be difficult and inaccurate in a range of delivery settings (Schorn 2010) . Furthermore, uncertainties remain about the quantities of blood loss that are of greatest importance in predicting severe maternal outcomes for women with excessive bleeding. Some clinical signs and symptoms (such as pallor, weakness, palpitation, tachycardia, confusion, oliguria, etc) have been correlated with blood loss and may be useful for triggering clinical intervention in the management of PPH (Bonnar 2000) . A systematic review of the literature assessing the relationship between clinical signs and symptoms and blood loss with maternal outcomes suggests that a shock index, a calculated variable of heart rate to systolic blood pressure, may be a useful marker for evaluating the severity of blood loss (Pacagnella 2013). Most of the studies included in the review report an association between heart rate, blood pressure and/or shock index with blood loss. However, few of these studies were conducted among obstetric populations, in which pregnancy-induced hemodynamic changes might lead to a different relationship between clinical signs and blood loss. Thus, further research is warranted to assess the relationship between shock index and severity of blood loss during the immediate postpartum period. Recent retrospectives studies that evaluated the relationship between severe PPH and vital signs suggest that elevated levels of shock index (e.g. SI ≥ 0.9 or ≥ 1.7) can help to identify women in need of urgent .

**Prophylactic Oxytocin IV Infusion vs. IM Injection in the Third Stage of Labor**

**Research Objectives**

Primary objectives

- To evaluate whether 10 IU prophylactic oxytocin administered in the third stage of labor via IV infusion results in a lower mean blood loss compared to IM injection.
- To also compare the effect of IV infusion and IM oxytocin administration on the proportion of women who experience blood loss ≥ 500 ml.

Secondary objectives

- To compare the effect of IV infusion and IM oxytocin administration on the proportion of women who experience blood loss ≥ 1000 ml, side effects, adverse events and change in hemoglobin pre- to post-delivery.
- To determine the relationship that exists between changes postpartum in shock index and the severity of bleeding and other PPH outcomes.

This study will provide answers to the following research questions:

1. Is administration of oxytocin by IV infusion more effective than IM administration of oxytocin at reducing post-partum blood loss when provided as part of AMTSL?
2. Is the mean difference between pre-delivery and post-delivery hemoglobin levels less with IV infusion than with IM administration?
3. Do the side effect profiles differ between IV infusion and IM oxytocin administration?

# Is shock index a potential useful indicator for evaluating the severity of postpartum bleeding during the first hour postpartum and initating clincial intervention for PPH management?

**Outcome Measures**

Primary outcome measures:

- Mean blood loss (ml)
- Proportion of women who bleed ≥ 500 ml

Secondary outcome measures:

- Proportion of women who bleed ≥ 1000 ml
- Change in hemoglobin from pre-delivery to postpartum
- Time to placental delivery
- Administration of additional oxytocin, other uterotonics, or other interventions such as blood transfusion and hysterectomy
- Side effects 1 hour postpartum
- Measurements of blood pressure and pulse during the first hour postpartum.

**Description of Study Site**

*Corrientes, Argentina:* El Hospital J.R. Vidal is a public, tertiary level institution situated in the capital of Corrientes. In 2012, a total of 3,080 deliveries were atended at this instituion. Approximately, 30% were cesarean deliveries. Oxytocin is available and used routinely during the third stage of labor.

**Explanation of Sample Size**

The primary outcomes of the study are (1) mean blood loss and (2) the proportion of women with PPH (defined as ≥500 ml).

This study is designed to detect a 50 ml difference in mean blood loss after provision of oxytocin prophylaxis via IV infusion and IM injection. In the published literature, a 50 ml difference has been shown to translate to approximately a 50% reduction in PPH rates (≥500 ml) . Based on the assumption that oxytocin prophylaxis IV will yield a mean blood loss of 275 (SD175)we estimate that a total of 257 women would be required in this superiority trial to detect a 50 ml difference in mean blood loss between the two groups with 80% power at a significance level of 0.05.

A prior study of PPH conducted at Hospital Vida had documented a rate of blood loss ≥500 ml of 18%, among women who received prophylactic oxytocin (10 IU) in the third stage of labor and with blood loss objectively assessed using a calibrated receptacle. Based on the assumption that oxytocin prophylaxis IV will yield a clinically meaningful reduction of at least 50% in the proportion of women with blood loss ≥500 ml , we calculate that a total of 442 women (vaginal deliveries) (221 per group) are required in this superiority trial to compare a 18% PPH rate following IM injection and a 9% PPH rate following IV infusion with 80% power at a significance level of 0.05. The sample size will be increased by 10% to account for any loss to follow up or missing blood loss readings for assessment of both primary outcomes. Thus, **a total sample size of 486 (243 per arm) is required** for the study in Ecuador. This sample size will be sufficient to detect a 50 ml mean difference in blood loss between study groups.

**Description of Research**

This double-blind, controlled and randomized study will be carried out in a hospital where policy dictates that all women should receive 10 IU of prophylactic oxytocin in the third stage of labor. Furthermore, the majority of women who give birth in the study hospital already have an IV line in place. The study objective will be to evaluate the impact of route of administration of 10 IU oxytocin on postpartum bleeding. Eligible women who provide informed consent and give birth vaginally will be randomized to receive either:

- 10 IU oxytocin via IV infusion
- 10 IU oxytocin via IM injection

Oxytocin 10 IU will be administered either by IV infusion or IM according to randomization assignment as soon as possible after delivery of the baby. The IV infusion will be done by placing 10 IU of oxytocin in a bag of saline solution of 500 cc and then administered over 40 minutes (eg, 12cc/min or 240drops/min). All study participants will receive oxytocin by IV infusion or by IM injection, based on randomized allocation, and the corresponding placebo by the non-assigned route.

To avoid any potential bias that knowledge of the route of administration of oxytocin may have in the quantification of blood loss, study staff members and participants will not know the assignment of the route of administration of the active drug. Since all delivering women in the study hospital routinely have an IV line in place, all study participants will receive oxytocin by either IV infusion or IM injection, based on randomization assignment, in addition to placebo by the non-assigned route. Blood loss will be measured in a calibrated container for 1 hour. If a woman experiences excessive bleeding or PPH, she will receive treatment according to standard of care at the study site.

Independent of the diagnosis of PPH, consenting women who have a vaginal delivery will have their postpartum blood loss measured using a standardized receptacle, in addition to other indicators and outcomes recorded during the immediate postpartum period to assess the relationship between shock index and PPH outcomes. Blood loss will be measured for 1 hour, and if PPH diagnosed, measurement will continue until cessation of active bleeding. Pulse and blood pressure will be measured at pre-defined intervals (15, 30, 45, and 60 minutes) in order to document hemodynamic changes that occur during the immediate postpartum period.

***Eligibility Criteria***

*Inclusion criteria*

All women who present in active labor for a live birth at the study hospital will be considered for participation in the study. Women who present for care for a vaginal delivery will be enrolled. Independent of being diagnosed with PPH, among women who provide their consent and who have vaginal delivery, postpartum blood loss will be collected and measured, as well as other clinical measures recorded in the immediate postpartum period in order to evaluate the relationship between the shock index and outcomes of PPH.

*Exclusion criteria*

Women who are:

- Planned or transferred for delivery via Cesarean section
- That reject having an IV line in place
- Unable to provide informed consent due to mental impairment, distress during labor or other reason
- Unwilling and/or unable to respond to questionnaires about background characteristics.

If at any point in time the woman wants to withdraw from the study, she will be dismissed and data collection will cease.

***Informed Consent and Pre-delivery Procedures***

Informed consent will be obtained by trained study personnel after women have been admitted to the hospital and upon arrival at the labor ward. If study personnel feel that the woman is too far advanced in her labor to give appropriate informed consent, she will not be eligible for enrollment. Women who decline to participate in the study will be given full medical care according to standard practice in the facility. Women who wish to participate will be asked to read and sign the informed consent form. If the woman is unable to read, the form will be read and explained to her, and her consent will be indicated by a mark, such as the woman’s thumbprint. If at any point during her participation in the study the woman indicates that she no longer wishes to continue, she will be withdrawn from further data collection.

Background characteristics and baseline data will be obtained and documented on Form 1. Pre-delivery hemoglobin will be measured and recorded for all women, using a Hemocue® Hemoglobin machine + cuvette (HemoCue, Ängelholm, Sweden).

***Randomization and Enrollment***

All participants will be randomized to prophylactic management with oxytocin in the third stage of labor either via IV infusion or IM injection, with the assignment contained in sequentially numbered sealed opaque envelopes. During the second stage, prior to each woman’s delivery, study personnel will open the next sequential randomization packet containing her study group assignment. Since the study will be blinded and placebo-controlled, study personnel will open the next sequential drug packet, which will contain a vial each of 10 IU oxytocin and 10 IU matching placebo. Each vial will have an affixed label indicating the assigned route of administration of the contents.

10 IU oxytocin ampoules and similar placebo ampoules (containing 10 IU of saline) will be prepared by a local laboratory / pharmacist. The ampoules prepared for the study (e.g. study packets) will have to be stored in a refrigerator to maintain the cold chain necessary for oxytocin until the time of administration to the mothers. The randomization code will be generated in advance by Gynuity Health Projects staff in New York. The random assignment will be made in blocks of 10 and will be generated with a computer program. Only the Gynuity study coordinator will have access to this randomization scheme. Once an administration assignment has been allocated, the woman will be considered an enrolled participant.

Study staff will observe women and will record any intervention, including other AMTSL components performed during delivery. In addition, staff will note whether a woman has had her labor induced or augmented prior to delivery and, if possible, the drug used and time administered. In the case of labor augmentation with oxytocin, it should also be noted that the agent was discontinued upon delivery of the fetal head.

***Intervention***

Oxytocin 10 IU will be administered either by IV infusion or IM according to randomization assignment as soon as possible after delivery of the baby.

Immediately following delivery of the baby (or last baby, in the case of multiples), blood loss will be measured in a calibrated container for 1 hour. Blood loss will be recorded when active bleeding stops or at 1 hour, whichever is later. In the case of excessive bleeding or PPH requiring treatment, blood loss will be documented also at time of diagnosis. At delivery, some blood can be expected to spatter on the drapes and on the gowns of the attendant, and all attempts will be made to minimize such losses. Additionally, the placental interstices contain maternal blood (about 9% of the placental weight). We expect that overestimations (amniotic fluid) and underestimations (blood loss) are likely to be distributed between the two study arms; therefore there will be no need for corrections to the blood measurement for these inaccuracies.

If a woman experiences excessive bleeding or PPH and requires treatment, she should receive treatment according to standard of care at the study site. If PPH is diagnosed, measurement will continue until the bleeding stops. Health providers can diagnose PPH at any time, but if blood loss reaches 500 ml according to the marking on the calibrated container, PPH should be diagnosed and standard hospital treatment initiated.

The study staff will keep the participants under observation and record all interventions performed during delivery, including the prophylactic oxytocin administration of the study. Also, note if the delivery was induced or stimulated before delivery and if possible, the medication provided, and the time and dose in which it was administered. The provider will be asked to monitor the woman for side effects for 1 hour after delivery. Women will also respond to questions about side effects prior to discharge from the hospital.

In order to document changes in shock index values during the first hour after delivery, the heart rate and blood pressure of all women enrolled at 15, 30, 45 and 60 minute intervals will be recorded. These measurements will be taken independently of the diagnosis of PPH and the exact time of each measurement will be recorded. Automatic devices will be provided to all study centers to measure blood pressure and heart rate in order to standardize such measurements.

Post-delivery hemoglobin using a Hemocue® Hemoglobin machine + cuvette will be taken at least 24 after delivery and before the woman leaves the facility. If the woman has received IV fluids, hemoglobin will be measured at least 12 hours after removal of the IV prior to discharge.

***Study Documentation***

*Admission*. This form will document basic demographic characteristics and medical and obstetrical history to assess women’s eligibility for study participation. It will also document that each woman has signed the informed consent form. This form will be completed for all consenting women.

*Randomization/Delivery*. This form will document randomization number and relevant information during and post delivery, including time of delivery and events or interventions during the third stage of labor, especially with regard to administration of uterotonic agents as well as other components of active management. This form will also record any diagnosis of PPH. The form will be completed for all consenting women.

*Follow-up.* This form will be used to record information on side effects, adverse events, or problems following delivery at follow-up prior to discharge from the hospital. Post-delivery hemoglobin will be measured and recorded at this time.

*Severe Adverse Events (SAEs) Form*. This form will be used to document any SAEs as defined below. It should be completed by the principal investigator and forwarded to Gynuity Health Projects staff within 24 hours of his/her awareness of the event.

A serious adverse event is defined as one causing:

- Prolonged hospitalization;
- Permanent or serious disability;
- Additional threat to life; or
- Death.

**Data Management and Monitoring**

Copies of all study forms for the first five women enrolled will be sent to the study coordinator at Gynuity Health Projects for review prior to continuation of enrollment. Thereafter, copies of the forms will be sent periodically to the Gynuity Health Projects in New York. All original forms will be retained at the study site.

Data will be entered into an SPSS database for subsequent review of logical inconsistencies, cleaning, and analysis.

***Data and Safety Monitoring***

Gynuity Health Projects will monitor the progress of each site participating in the study. Trained monitors will visit sites a minimum of two times during the study to observe practices and review documentation to verify that:

- The rights and well-being of human subjects are protected;
- Data are collected according to the protocol, are accurate, complete and of the highest quality and integrity;
- Trial is in compliance with the approved protocol and Good Clinical Practices.

Monitors will pay particular attention to ensure that the women recruited to participate in the study fulfill eligibility criteria, enrollment is progressing adequately, informed consent is appropriately documented, providers adhere to the randomization scheme, data are appropriately reported, and protocol violations/deviations are avoided. Any deviations from the protocol or practices that elicit concern on the part of the monitor will be discussed with the sites’ Investigator(s) and corrective measures will be taken.

***Analysis and Dissemination of Results***

Bivariate analyses will be conducted, stratified by study arm. Unadjusted and adjusted logistic regression will be conducted respectively for categorical and continuous dependent variables.

To compare the two study arms (oxytocin IM vs. oxytocin infusion), the main analyses will include:

- Mean postpartum blood loss (primary outcome)
- % of women with blood loss ≥500 ml (primary outcome)
- Median postpartum blood loss and interquartile range
- % of women with blood loss ≥1000 ml,
- Mean change in Hb measured pre- to post-delivery
- % of women with postpartum Hb drops of 2 g/dl or greater
- % of women with prolonged third stage of labor
- % of women who received additional oxytocics or other interventions to manage bleeding
- % of women who experienced side effects

We will register use of any other AMTSL components, such as controlled cord traction, uterine massage, etc. that are used.

In order to assess the relationship between shock index and severity of bleeding, we will correlate shock index values, heart rate, and blood pressure measures to blood loss levels that will be recorded at 15, 30, 45, and 60 minutes following delivery. Absolute values and measured changes in these values during the first hour following delivery will be analyzed. We will also explore associations between these clinical signs/symptoms with other PPH outcomes, including provision of additional interventions to control bleeding, blood transfusion, and other severe outcomes.

Data will be owned by Gynuity Health Projects. The principal investigators and study coordinators will work together to analyze the results of the study and prepare at least one manuscript for submission to one or more relevant peer-reviewed journals. In addition, members of the study team will present the results of the study at conferences and meetings where appropriate. All principal investigators will be named along with key Gynuity staff as authors of any publications or presentations describing results from the study.

**Human Subjects**

The study will be conducted in accordance with the current version of the Declaration of Helsinki. The protocol requires that an informed consent must be available for all study participants. The protocol must be approved by Institutional Review Board/Ethical Committee before the first study participant is enrolled.

All study documents and participant records will be assigned unique identifiers comprised of participant initials and a numerical code. Women’s names will not be associated with study documents to maintain participant confidentiality. All records will be kept in a locked filing cabinet and only authorized study personnel will have access to these files. Clinical information will not be released without the permission of the participant, except as required for monitoring.

**References**

1. WHO, *WHO recommendations for the prevention of postpartum haemorrhage.* WHO, 2007.

2. Midwives;, I.C.o. and I.F.o.G.a. Obstetrics, *Prevention and Treatment of Post-partum haemorrhage: New Advances for Low Resource Settings.* 2006: p. 1-4.

3. Cotter, A.M., Ness Amen, and J.E. Tolosa, *Prophylactic oxytocin for the third stage of labour.* Cochrane Database Systems Review; John Wiley & Sons, Ltd., 2001(4).

4. Prendiville, W.J., et al., *The Bristol third stage trial: active versus physiological management of third stage of labour.* BMJ, 1988. **297**(6659): p. 1295-300.

5. de Groot, A.N.J.A., et al., *A placebo-controlled trial of oral ergometrine to reduce postpartum hemorrhage.* Acta Obstetricia et Gynecologica Scandinavica, 1996. **75**(5): p. 464 - 468.

6. Nordstrom, L., et al., *Routine oxytocin in the third stage of labour: a placebo controlled randomised trial.* British Journal of Obstetrics and Gynaecology, 1997. **104**(7): p. 781-6.

7. Pierre, F., L. Mesnard, and G. Body, *For a systematic policy of i.v. oxytocin inducted placenta deliveries in a unit where a fairly active management of third stage of labour is yet applied: results of a controlled trial.* European Journal of Obstetrics, Gynecology, and Reproductive Biology, 1992. **43**(2): p. 131-5.

8. Choy, C.M., et al., *A randomised controlled trial of intramuscular syntometrine and intravenous oxytocin in the management of the third stage of labour.* International Journal of Gynecology and Obstetrics, 2002. **109**(2): p. 173-7.

9. Rashid, M., A. Clark, and M.H. Rashid, *A randomised controlled trial comparing the efficacy of intramuscular syntometrine and intravenous syntocinon, in preventing postpartum haemorrhage.* J Obstet Gynaecol, 2009. **29**(5): p. 396-401.

10. Ilancheran, A. and S.S. Ratnam, *Effect of oxytocics on prostaglandin levels in the third stage of labour.* Gynecol Obstet Invest, 1990. **29**(3): p. 177-80.

11. Fugo, N.W. and W.J. Dieckmann, *A comparison of oxytocic drugs in the management of the placental stage.* Am J Obstet Gynecol, 1958. **76**(1): p. 141-6.

12. Sorbe, B., *Active pharmacologic management of the third stage of labor. A comparison of oxytocin and ergometrine.* Obstet Gynecol, 1978. **52**(6): p. 694-7.

13. Howard, W.F., P.R. McFadden, and W.C. Keettel, *Oxytocic Drugs in Fourth Stage of Labor.* Jama, 1964. **189**: p. 411-3.

14. Tsu, V.D., et al., *Reducing postpartum hemorrhage in Vietnam: assessing the effectiveness of active management of third-stage labor.* J Obstet Gynaecol Res, 2006. **32**(5): p. 489-96.

15. Poeschmann, R.P., W.H. Doesburg, and T.K. Eskes, *A randomized comparison of oxytocin, sulprostone and placebo in the management of the third stage of labour.* Br J Obstet Gynaecol, 1991. **98**(6): p. 528-30.

16. Newton, M., et al., *Blood loss during and immediately after delivery.* Obstet Gynecol, 1961. **17**: p. 9-18.

17. Gulmezoglu, A.M., et al., *WHO multicentre randomised trial of misoprostol in the management of the third stage of labour.* Lancet, 2001. **358**(9283): p. 689-95.

18. Gibbens, D., et al., *The circulating levels of oxytocin following intravenous and intramuscular administration of Syntometrine.* J Obstet Gynaecol Br Commonw, 1972. **79**(7): p. 644-6.

19. Lyndon A, L.D., Shields L, Melsop K, Bingham B, Main E (Eds). *Improving Health Care Response to Obstetric Hemorrhage. (California Maternal Quality Care Collaborative Toolkit to Transform Maternity Care)*, in *Improving Health Care Response to Obstetric Hemorrhage*. 2010, California Maternal Quality Care Collaborative.

20. Crall, H.D. and D.R. Mattison, *Oxytocin pharmacodynamics: effect of long infusions on uterine activity.* Gynecol Obstet Invest, 1991. **31**(1): p. 17-22.

21. Cunningham, F.G. and J.W. Williams, *Williams obstetrics*. 21st ed. 2001, New York: McGraw-Hill. x, 1668 p.

22. Gonser, M. and P. Kahle, *Estimated linear relationship between ductus venosus index and gestational age.* Ultrasound Obstet Gynecol, 1994. **4**(1): p. 85; author reply 86.

23. Prendiville, W.O.C., M., *Active Management of the Third Stage of Labor*. 1 ed. A Textbook of Postpartum Hemorrhage, ed. C.B.-L.L.G.K.A.B.L.M. Karoshi. 2006, Duncow: Sapiens Publishing. 462.

24. Seitchik, J., et al., *Oxytocin augmentation of dysfunctional labor. IV. Oxytocin pharmacokinetics.* Am J Obstet Gynecol, 1984. **150**(3): p. 225-8.

25. Gonser, M., *Labor induction and augmentation with oxytocin: pharmacokinetic considerations.* Arch Gynecol Obstet, 1995. **256**(2): p. 63-6.

26. Huh, W.K., D. Chelmow, and F.D. Malone, *A Double-Blinded, Randomized Controlled Trial of Oxytocin at the Beginning versus the End of the Third Stage of Labor for Prevention of Postpartum Hemorrhage.* Gynecologic and Obstetric Investigation, 2004. **58**(2): p. 72-6.

27. Soriano, D., et al., *A prospective cohort study of oxytocin plus ergometrine compared with oxytocin alone for prevention of postpartum haemorrhage.* Br J Obstet Gynaecol, 1996. **103**(11): p. 1068-73.

28. Villar, J., et al., *Systematic review of randomized controlled trials of misoprostol to prevent postpartum hemorrhage.* Obstetrics and Gynecology, 2002. **100**(6): p. 1301-12.

29. Sheldon, W., *How effective are the components of active management of the third stage of labor?*, in *XXII Asian and Oceanic Congress of Obstetrics and Gynecology*. 2011, Gynuity Health Projects: Taipei, Taiwan. .

30. Davies, G.A., et al., *Maternal hemodynamics after oxytocin bolus compared with infusion in the third stage of labor: a randomized controlled trial.* Obstet Gynecol, 2005. **105**(2): p. 294-9.

31. SICOR Pharmaceuticals, I., *Oxytocin Injection, USP Synthetic 10 units/1 mL*, FDA, Editor. 2008: Irvine.

1. WHO, *WHO recommendations for the prevention of postpartum haemorrhage.* WHO, 2007.

2. Midwives;, I.C.o. and I.F.o.G.a. Obstetrics, *Prevention and Treatment of Post-partum haemorrhage: New Advances for Low Resource Settings.* 2006: p. 1-4.

3. Cotter, A.M., Ness Amen, and J.E. Tolosa, *Prophylactic oxytocin for the third stage of labour.* Cochrane Database Systems Review; John Wiley & Sons, Ltd., 2001(4).

4. Prendiville, W.J., et al., *The Bristol third stage trial: active versus physiological management of third stage of labour.* BMJ, 1988. **297**(6659): p. 1295-300.

5. de Groot, A.N.J.A., et al., *A placebo-controlled trial of oral ergometrine to reduce postpartum hemorrhage.* Acta Obstetricia et Gynecologica Scandinavica, 1996. **75**(5): p. 464 - 468.

6. Nordstrom, L., et al., *Routine oxytocin in the third stage of labour: a placebo controlled randomised trial.* British Journal of Obstetrics and Gynaecology, 1997. **104**(7): p. 781-6.

7. Pierre, F., L. Mesnard, and G. Body, *For a systematic policy of i.v. oxytocin inducted placenta deliveries in a unit where a fairly active management of third stage of labour is yet applied: results of a controlled trial.* European Journal of Obstetrics, Gynecology, and Reproductive Biology, 1992. **43**(2): p. 131-5.

8. Choy, C.M., et al., *A randomised controlled trial of intramuscular syntometrine and intravenous oxytocin in the management of the third stage of labour.* International Journal of Gynecology and Obstetrics, 2002. **109**(2): p. 173-7.

9. Rashid, M., A. Clark, and M.H. Rashid, *A randomised controlled trial comparing the efficacy of intramuscular syntometrine and intravenous syntocinon, in preventing postpartum haemorrhage.* J Obstet Gynaecol, 2009. **29**(5): p. 396-401.

10. Ilancheran, A. and S.S. Ratnam, *Effect of oxytocics on prostaglandin levels in the third stage of labour.* Gynecol Obstet Invest, 1990. **29**(3): p. 177-80.

11. Fugo, N.W. and W.J. Dieckmann, *A comparison of oxytocic drugs in the management of the placental stage.* Am J Obstet Gynecol, 1958. **76**(1): p. 141-6.

12. Sorbe, B., *Active pharmacologic management of the third stage of labor. A comparison of oxytocin and ergometrine.* Obstet Gynecol, 1978. **52**(6): p. 694-7.

13. Howard, W.F., P.R. McFadden, and W.C. Keettel, *Oxytocic Drugs in Fourth Stage of Labor.* JAMA, 1964. **189**: p. 411-3.

14. Tsu, V.D., et al., *Reducing postpartum hemorrhage in Vietnam: assessing the effectiveness of active management of third-stage labor.* J Obstet Gynaecol Res, 2006. **32**(5): p. 489-96.

15. Poeschmann, R.P., W.H. Doesburg, and T.K. Eskes, *A randomized comparison of oxytocin, sulprostone and placebo in the management of the third stage of labour.* Br J Obstet Gynaecol, 1991. **98**(6): p. 528-30.

16. Newton, M., et al., *Blood loss during and immediately after delivery.* Obstet Gynecol, 1961. **17**: p. 9-18.

17. Gulmezoglu, A.M., et al., *WHO multicentre randomised trial of misoprostol in the management of the third stage of labour.* Lancet, 2001. **358**(9283): p. 689-95.

18. Gibbens, D., et al., *The circulating levels of oxytocin following intravenous and intramuscular administration of Syntometrine.* J Obstet Gynaecol Br Commonw, 1972. **79**(7): p. 644-6.

19. Lyndon A, L.D., Shields L, Melsop K, Bingham B, Main E (Eds). *Improving Health Care Response to Obstetric Hemorrhage. (California Maternal Quality Care Collaborative Toolkit to Transform Maternity Care)*, in *Improving Health Care Response to Obstetric Hemorrhage*. 2010, California Maternal Quality Care Collaborative,.

20. Crall, H.D. and D.R. Mattison, *Oxytocin pharmacodynamics: effect of long infusions on uterine activity.* Gynecol Obstet Invest, 1991. **31**(1): p. 17-22.

21. Cunningham, F.G. and J.W. Williams, *Williams obstetrics*. 21st ed. 2001, New York: McGraw-Hill. x, 1668 p.

22. Gonser, M. and P. Kahle, *Estimated linear relationship between ductus venosus index and gestational age.* Ultrasound Obstet Gynecol, 1994. **4**(1): p. 85; author reply 86.

23. Prendiville, W.O.C., M., *Active Management of the Third Stage of Labor*. 1 ed. A Textbook of Postpartum Hemorrhage, ed. C.B.-L.L.G.K.A.B.L.M. Karoshi. 2006, Duncow: Sapiens Publishing. 462.

24. Seitchik, J., et al., *Oxytocin augmentation of dysfunctional labor. IV. Oxytocin pharmacokinetics.* Am J Obstet Gynecol, 1984. **150**(3): p. 225-8.

25. Gonser, M., *Labor induction and augmentation with oxytocin: pharmacokinetic considerations.* Arch Gynecol Obstet, 1995. **256**(2): p. 63-6.

26. Huh, W.K., D. Chelmow, and F.D. Malone, *A Double-Blinded, Randomized Controlled Trial of Oxytocin at the Beginning versus the End of the Third Stage of Labor for Prevention of Postpartum Hemorrhage.* Gynecologic and Obstetric Investigation, 2004. **58**(2): p. 72-6.

27. Soriano, D., et al., *A prospective cohort study of oxytocin plus ergometrine compared with oxytocin alone for prevention of postpartum haemorrhage.* Br J Obstet Gynaecol, 1996. **103**(11): p. 1068-73.

28. Villar, J., et al., *Systematic review of randomized controlled trials of misoprostol to prevent postpartum hemorrhage.* Obstetrics and Gynecology, 2002. **100**(6): p. 1301-12.

29. Sheldon, W.R.D., Jill; Winikoff, Beverly; Blum, Jennifer; Trussell, James, *How effective are the components of active management of the third stage of labor?* BMC Pregnancy and Childbirth, 2013. **13**(46).

30. Davies, G.A., et al., *Maternal hemodynamics after oxytocin bolus compared with infusion in the third stage of labor: a randomized controlled trial.* Obstet Gynecol, 2005. **105**(2): p. 294-9.

31. SICOR Pharmaceuticals, I., *Oxytocin Injection, USP Synthetic 10 units/1 mL*, FDA, Editor. 2008: Irvine.

32. MN, S., *Measurement of blood loss: review of the literature.* J Midwifery Wom Heal, 2010. **55**(1): p. 20-27.

33. Bonnar, J., *Massive obstetric haemorrhage.* Baillieres Best Pract Res Clin Obstet Gynaecol, 2000. **14**(1): p. 1-18.

34. Pacagnella, R.C., et al., *A systematic review of the relationship between blood loss and clinical signs.* PLoS One, 2013. **8**(3): p. e57594.

35. Le Bas, A., et al., *Use of the "obstetric shock index" as an adjunct in identifying significant blood loss in patients with massive postpartum hemorrhage.* Int J Gynaecol Obstet, 2014. **124**(3): p. 153-5.

36. Nathan, H., et al., *Shock index: an effective predictor of outcome in postpartum haemorrhage?* BJOG, 2015. **122**(2): p. 268-75.

37. El Ayadi, A.M., et al., *Vital Sign Prediction of Adverse Maternal Outcomes in Women with Hypovolemic Shock: The Role of Shock Index.* PLoS One, 2016. **11**(2): p. e0148729.

38. Khan, G.Q., et al., *Controlled cord traction versus minimal intervention techniques in delivery of the placenta: a randomized controlled trial.* Am J Obstet Gynecol, 1997. **177**(4): p. 770-4.

39. Derman, R.J., et al., *Oral Misoprostol in preventing postpartum haemorrhage in resource-poor communities: a randomised controlled trial.* The Lancet, 2006. **368**(9543): p. 1248-53.

40. Dzuba, I., *Reduciendo el sangrado postparto: Uterotónicos profilácticos y consideraciones para su uso* in *XXI Congreso FLASOG*. 2014: Guayaquil, Ecuador.

41. Anger, H., *Route of Administration of Oxytocin in Prevention of Postpartum Hemorrhage*, in *XXI FIGO World Congress of Gynecology and Obstetrics*. 2015: Vancouver, Canada.
